# Influence of Tryptophan Contained in 1-Methyl-Tryptophan on Antimicrobial and Immunoregulatory Functions of Indoleamine 2,3-Dioxygenase

**DOI:** 10.1371/journal.pone.0044797

**Published:** 2012-09-13

**Authors:** Silvia K. Schmidt, Stephan Siepmann, Katja Kuhlmann, Helmut E. Meyer, Sabine Metzger, Sabine Pudelko, Margret Leineweber, Walter Däubener

**Affiliations:** 1 Institute of Medical Microbiology and Hospital Hygiene, Heinrich-Heine-University Düsseldorf, Düsseldorf, Germany; 2 Medical Proteome Center, Ruhr-University Bochum, Bochum, Germany; 3 Biological-Medical Research Center, Heinrich-Heine-University Düsseldorf, Germany; University of New South Wales, Australia

## Abstract

Indoleamine 2,3-dioxygenase (IDO) has been identified as an important antimicrobial and immunoregulatory effector molecule essential for the establishment of tolerance by regulating local tryptophan (Trp) concentrations. On the other hand, the immunosuppressive capacity of IDO can have detrimental effects for the host as it can lead to deleterious alterations of the immune response by promoting tolerance to some types of tumors. To suppress this disadvantageous IDO effect, the competitive inhibitor 1-Methyl-Tryptophan (1-MT) is being tested in clinical trials. However, it remains inconclusive which stereoisomer of 1-MT is the more effective inhibitor of IDO-mediated immunosuppression. While IDO enzyme activity is more efficiently inhibited by 1-L-MT in cell-free or *in vitro* settings, 1-D-MT is superior to 1-L-MT in the enhancement of anti-tumor responses *in vivo*.

Here, we present new data showing that commercially available 1-L-MT lots contain tryptophan in amounts sufficient to compensate for the IDO-mediated tryptophan depletion *in vitro*. The addition of 1-L-MT abrogated IDO-mediated antimicrobial effects and permitted the growth of the tryptophan-auxotroph microorganisms *Staphylococcus aureus* and *Toxoplasma gondii.* Consistent with this, the tryptophan within 1-L-MT lots was sufficient to antagonize IDO-mediated inhibition of T cell responses. Mass spectrometry (MS) analysis revealed not only tryptophan within 1-L-MT, but also the incorporation of this tryptophan in bacterial and human proteins that were generated in the presence of 1-L-MT in otherwise tryptophan-free conditions. In summary, these data reveal that tryptophan within 1-L-MT can affect the results of *in vitro* studies in an L-stereospecific and IDO-independent way.

## Introduction

L-tryptophan is an essential amino acid that is a prerequisite for protein biosynthesis and the production of important hormones like melatonin and serotonin [1]. Nevertheless, most tryptophan, which is taken up with the food, is preferentially degraded via the kynurenine pathway. The resulting kynurenine is subsequently discarded via the urine, or gradually processed to a number of biologically active metabolites. In mammals, the first and rate-limiting step of the kynurenine pathway can be executed by three enzymes: tryptophan 2,3-dioxygenase (TDO), indoleamine 2,3-dioxygenase (IDO) and indoleamine 2,3-dioxygenase-2 (IDO-2). All three enzymes are dioxygenases that can oxidize tryptophan to *N*-formylkynurenine.

On a functional level the activity of IDO has been most intensively analyzed. IDO is an immunoregulated enzyme and its ability to reduce local tryptophan concentrations induces bacteriostasis [2]–[4]. Furthermore, tryptophan depletion caused by IDO inhibits T-cell activation [5], [6], and kynurenine, the product of tryptophan catabolism, regulates T-cell growth and survival [7]. Additionally, IDO has been shown to play an important role in tumor-induced tolerance [8]. IDO expression has been observed in primary tumors and human tumor-draining lymph nodes in proximity to CD4^+^CD25^+^ regulatory T cells (Tregs), and studies with mice have revealed an IDO-dependent differentiation of naïve CD4^+^ T cells into Foxp3^+^ Tregs [9].

In 1991 Cady and Sono showed that 1-DL-Methyl-tryptophan (1-MT) is a competitive inhibitor of the rabbit small intestinal IDO [10]. Since then various groups have described the inhibition of IDO activity by 1-MT in several species. It has been observed that the antimicrobial [11], as well as the immunoregulatory, function of IDO [11]–[13] were abrogated by the use of this inhibitor.

Nevertheless, over the years contradictory results have also been generated when the two 1-MT enantiomers were analyzed separately for their capacity to inhibit IDO. For example, it has been shown that on the one hand the L isomer, but not the D isomer of 1-MT, was able to reverse the IDO-mediated arrest of T cell proliferation in several human *in vitro* model systems using different cell lines or cancer cells [13]. Furthermore, IDO activity in protein isolates of primary human colon cancer and interferon (IFN)-γ treated HeLa cells was inhibited by 1-L-MT only [14], [15]. On the other hand the D isomer of 1-MT was significantly more effective in *in vivo* antitumor responses, reversing the suppression of T cells mediated by IDO-positive human monocyte-derived or murine dendritic cells (DC) isolated from tumor-draining lymph nodes [16].

Overall, it has been shown that while 1-L-Methyl-tryptophan (1-L-MT) is a strong inhibitor of IDO in biochemical-based *in vitro* assays, 1-D-Methyl-tryptophan (1-D-MT) represses IDO-induced tumor growth *in vivo*. These strikingly conflicting findings were to some extent ascribed to the IDO-related enzyme IDO-2, also expressed in several human tumors [14] and found to be effectively inhibited by 1-D-MT [17]. However, studies in IDO-deficient mice and in human DCs after inhibition of IDO, revealed that the antitumor effect of 1-D-MT is a result of IDO targeting and not an off-target effect. This was shown by the fact that 1-D-MT lost its antitumor effect in IDO-deficient mice and that HeLa cells treated with IDO-specific siRNA completely lost their ability to degrade tryptophan [16], [14].

Here, we provide evidence for a new mode of action of 1-MT that is stereospecific for 1-L-MT. We reason that 1-L-MT contains tryptophan in concentrations permitting the growth of tryptophan-auxotroph bacteria (e.g. *Staphylococcus aureus*), in otherwise tryptophan-free medium. Similarly, 1-L-MT supplementation of tryptophan-free medium reinstitutes the proliferation of the parasite *Toxoplasma gondii*. Therefore, antimicrobial effects caused by IDO-dependent tryptophan depletion *in vitro*, can be abrogated by the addition of 1-L-MT in an IDO-independent fashion. Importantly, the immunoregulatory effects of IDO are also reinstated by the tryptophan contained in 1-L-MT, as human T cells proliferate in conditioned supernatants derived from IDO-positive tumor cells with the addition of 1-L-MT.

## Results

### 1-L-MT abrogates the IDO-mediated antibacterial effect in HeLa cells

**Figure 1:**
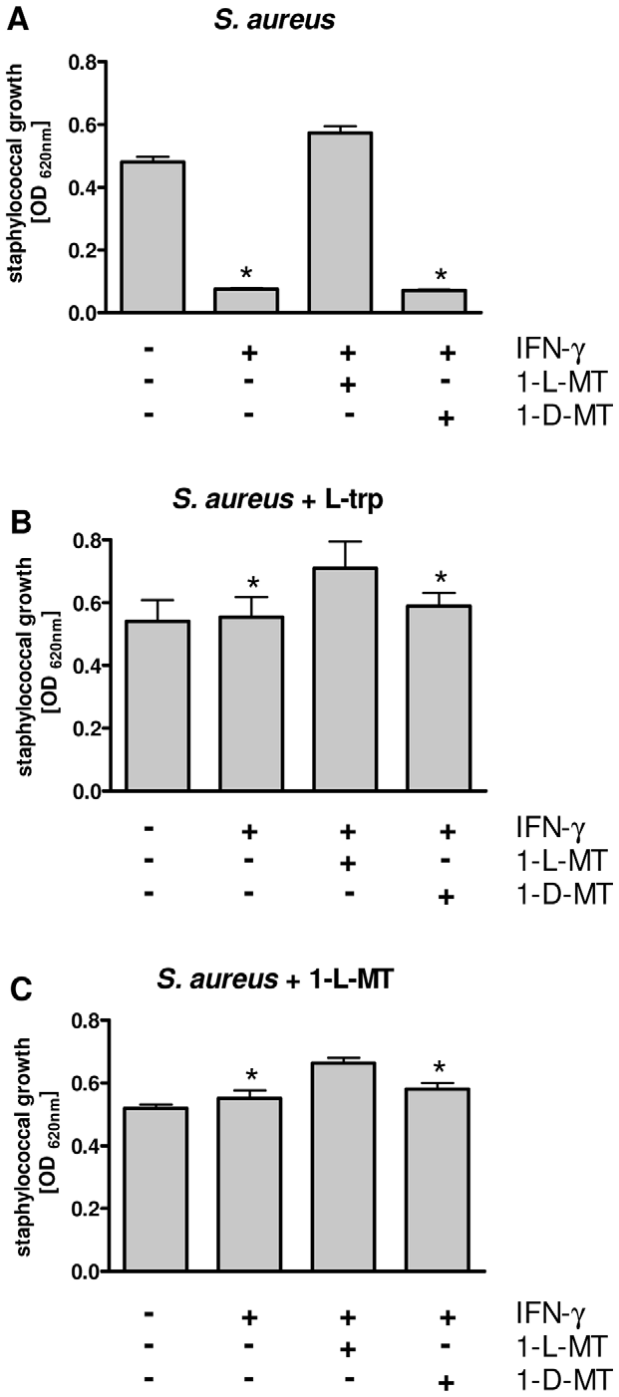
1-L-MT treatment abolishes the IDO-mediated antibacterial effect. HeLa cells were left unstimulated or stimulated with IFN-γ (500 U/mL) for 72 h. IFN-γ-stimulated cells were additionally treated with 1-L-MT or 1-D-MT (330 µg/mL each). Thereafter, cell supernatants were infected with *S. aureus* and bacterial growth was detected after additional 24 h (A) Cell culture supernatants were infected with bacteria and bacterial growth was determined photometrically. (B) The addition of L-tryptophan (L-trp) or 1-L-MT (C) to the supernatants at the time point of infection allowed bacterial growth in all experimental groups. Data are given as bacterial growth, determined by optical density at 620 nm, +/− SEM of three independent experiments with each experiment performed in triplicates. Asterisks indicate significant inhibition of bacterial growth (p<0.05).

IFN-γ stimulation of human cells promotes IDO activity, leading to a depletion of L-tryptophan and subsequent inhibition of bacterial growth [11], [15]. Figure 1A shows this antibacterial effect in conditioned medium harvested from IFN-γ-stimulated HeLa cells. *Staphylococcus aureus* (*S. aureus*) was able to grow in the conditioned medium of unstimulated controls, but the bacterial growth was inhibited in supernatants of stimulated cells. This IDO-mediated antibacterial effect could be blocked by the presence of the IDO inhibitor 1-L-MT during stimulation of HeLa cells, in comparison to 1-D-MT (330 µg/mL each), which did not abrogate the antibacterial effect of IDO stimulation. The observed bacteriostasis was proven to be due to the depletion of tryptophan, since the staphylococcal growth was restored by L-tryptophan (310 µg/mL) added at the time point of infection (Figure 1B). Interestingly, a simultaneous addition of 1-L-MT (330 µg/mL) together with the bacteria to the conditioned media also promoted bacterial growth (Figure 1C). Here, an effect of 1-L-MT on IDO activity was implausible because conditioned medium, lacking any IDO-expressing cells, served as culture medium for the staphylococci.

**Figure 2:**
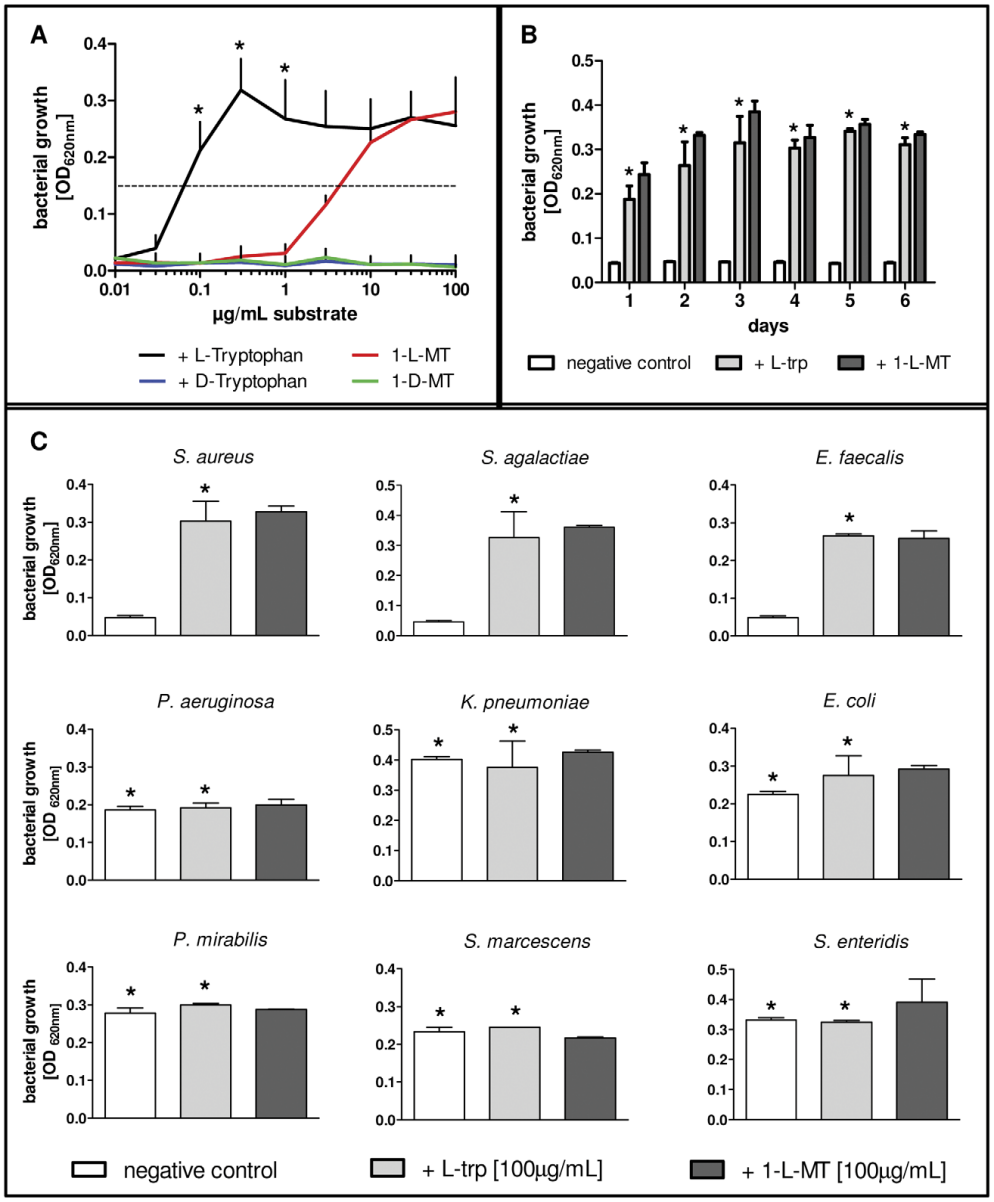
Bacterial growth in tryptophan-free medium with additional L-tryptophan or 1-L-MT. (A) *Staphylococcus aureus* was cultured in medium with denoted supplementation of L-trp (black), D-trp (blue), 1-L-MT (red) or 1-D-MT (green) (100 µg/mL each). After 18 h, bacterial growth was determined photometrically. Asterisks indicate significant increase of bacterial growth compared to the 1-L-MT group (p<0.05). (B) Long-term culture of *Staphylococcus aureus*. Bacteria were cultured within tryptophan-free culture medium with or without L-tryptophan or 1-L-MT (100 µg/mL each) and bacterial growth within each culture was determined after 18 h photometrically. Thereafter, the bacterial culture was diluted in PBS serially and 10 µl were used to infect new tryptophan-free culture medium, according to 10–100 CFU/well. Asterisks mark no significant difference (p<0.05) in the 1-MT group, compared to tryptophan. (C) Different bacterial strains were grown in tryptophan-free medium with additional L-tryptophan or 1-L-MT (100 µg/mL each) for 18 h. Asterisks mark no significant difference (p<0.05) in the 1-MT group, compared to tryptophan.. All data are given as bacterial growth, determined by optical density at 620 nm, +/− SEM of triplicates of three independent experiments (A) or +/− SD of duplicates of one representative experiment (B, C).

To exclude involvement of IDO activity and to investigate an effect of 1-L-MT on the bacteria themselves, *S. aureus* was cultured in custom-made, tryptophan-free culture medium supplemented with L-tryptophan or 1-L-MT. As expected, the bacteria were unable to grow in this culture medium, unless supplemented with L-tryptophan or with 1-L-MT (Figure 2A). In both groups the bacteria grew in a concentration-dependent manner: whereas only 0.05 µg/mL L-trp permitted half-maximal bacterial growth, 3 µg/mL of the 1-L-MT was required. An unspecific toxicity or a negative impact on the overall bacterial growth of 1-L-MT was ruled out by long-term growth experiments with *S. aureus* cultured in L-tryptophan or 1-L-MT supplemented tryptophan-free media (Figure 2B). Furthermore, the 1-L-MT-dependent growth was not a characteristic of the *S. aureus* strain used. 1-L-MT promoted also growth of other tryptophan-auxotroph bacteria such as *Streptococcus agalactiae or Enterococcus faecalis*. Accordingly, gram-negative rods (e.g. *Echerichia coli*, *Pseudomonas aeruginosa*) that are tryptophan prototrophic were not influenced by tryptophan-deficiency and therefore grew irrespective of the presence or absence of tryptophan or 1-L-MT (Figure 2C).

In summary, 1-L-MT treatment showed effects that were independent of its role as an IDO-specific inhibitor. 1-L-MT, but not 1-D-MT, was able to serve as a tryptophan substitute that permitted bacterial growth in the absence of a natural tryptophan source.

### Commercially available 1-L-MT contains proteinogenic L-tryptophan

**Figure 3:**
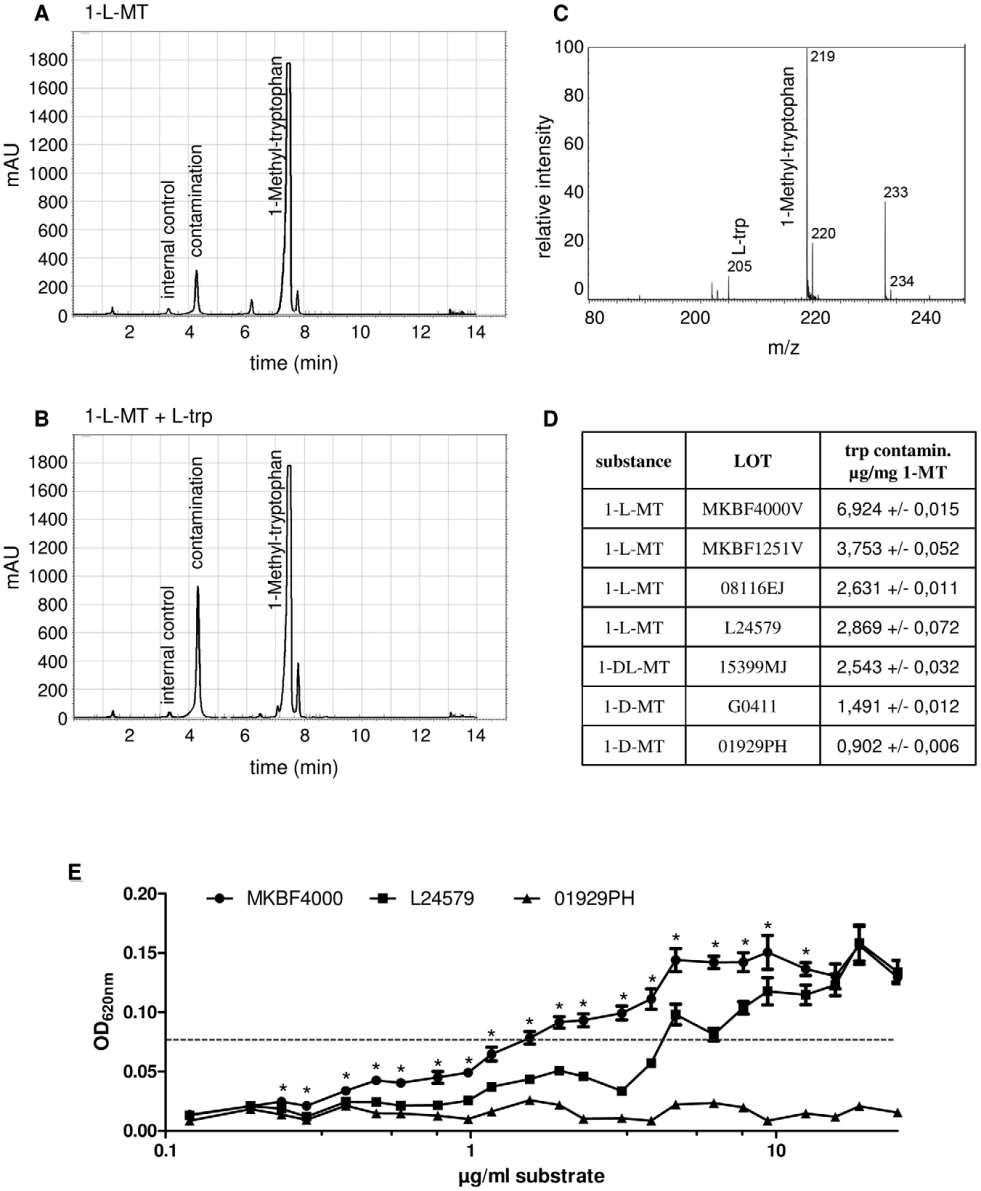
Analysis of 1-L-MT dissolved in tryptophan-free cell culture medium. (A) Exemplary HPLC chromatogram (lot L24579) showing 1-L-MT peak (at retention time 7.6 min) and “contamination” peak (at retention time 4.2 min). (B) Exemplary HPLC chromatogram of 1-L-MT lot L24579 with supplemented L-tryptophan (L-trp). Note that the L-trp peak appears at the same retention time as the “contamination” peak in A. For both analyses 3-nitrotyrosine (retention time 3.5 min) served as internal control. (C) MS analysis of 1-L-MT lot L24579 dissolved in tryptophan-free cell culture medium. Additional to 1-L-MT with the m/z of 219, also tryptophan with the m/z of 205 was detected. n = 1. (D) Table showing the tryptophan contamination (µg/mg 1-MT) in different 1-MT lots. (E) Diagram revealing the growth of *Staphylococcus aureus* in tryptophan-free medium in presence of different 1-MT lots. Asterisks indicate that the bacterial growth is significantly higher in the presence of MKBF4000V as compared to L24579.

We next chose to explore whether the unexpected growth of *S. aureus* in 1-L-MT treated supernatants resulted from an L-trp source in 1-L-MT or the utilisation of 1-L-MT itself. According to the manufacturer's instructions 1-MT has a purity of at least 95% (see material and method section for details). Nevertheless, it was not obvious if the 5% impurity comprised traces of tryptophan. To test this, HPLC analyses were performed. Various commercially available 1-L-MT lots were dissolved in custom-made, tryptophan-free RPMI cell culture medium and the potential contaminants were analyzed. 1-L-MT was stable for the observed time and could be detected at a retention time of 7.6 minutes (exemplary chromatogram Figure 3A). However, there was also a peak at a retention time of 4.2 minutes, corresponding to the tryptophan supplemented control sample (Figure 3B). 1-L-MT has the expected mass-to-charge-ratio (m/z) of 219, while the m/z of tryptophan is 205. MALDI-MS analysis revealed that a 205 peak was present in all analyzed 1-L-MT lots (Figure 3C and Supporting Information Figure S1). However, there were quantitative differences in the detection rate. Furthermore, we confirmed that the detected signal at m/z 205 represents tryptophan by performing ESI-MS/MS analysis and comparing the fragmentation patterns (data not shown). A detailed comparison between different 1-L-MT lots via HPLC analysis revealed that tryptophan was present in all samples, although the absolute amounts differed strongly. For example, the lot MKBF4000V contained about 2.5 times more tryptophan (6,924 µg tryptophan per mg 1-MT) than lot L24579 (2,869 µg tryptophan per mg 1-MT) (Figure 3D). Accordingly, bacteria needed 2.2 times more 1-L-MT for a half maximal growth (1,2 µg/mL in MKBF4000V and 2,69 µg/mL in L24579, respectively) (Figure 3E). Again, staphylococci were not able to grow in the presence of 1-D-MT (Figure 3E), although 1-D-MT was contaminated with D-tryptophan (Figure 3D).

**Figure 4:**
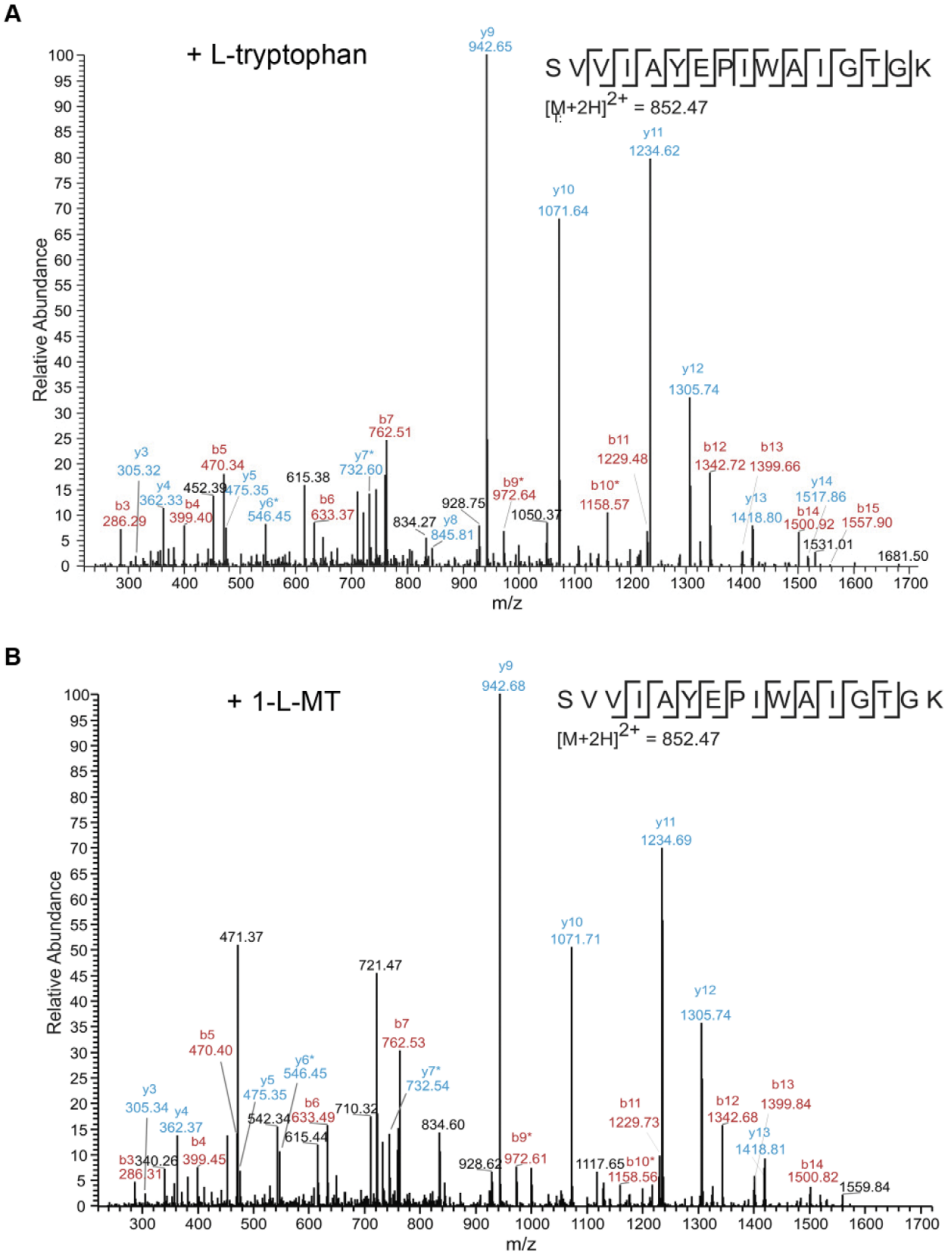
MS/MS spectra for a tryptophan-containing peptide from *S. aureus* cell lysate. *S. aureus* was grown in the presence of L-tryptophan (A) or 1-L-MT (B). From the spectra shown, the peptide SVVIAYEPIWAIGTGK from *S. aureus* Triosephosphate isomerase (Uniprot ID: A7WZS8) was identified with a mascot score of 63 (L-tryptophan) and 47 (1-L-MT). The peptide contains L-Tryptophan and not 1-L-MT in both samples. Fragment ions identifying the tryptophan residue are clearly visible and are marked with an asterisk.

Next we wanted to investigate, if 1-L-MT was proteinogenic itself. Bacteria (*Staphylococcus aureus*, *Streptococcus agalactiae*) cultivated in tryptophan-free medium could not grow unless supplemented with either L-tryptophan or 1-L-MT (100 µg/mL) (Figure 2A). After culture in these media bacteria were lysed and the bacterial proteins were analyzed via mass spectrometry (LC-MS/MS). From each sample, several hundred peptides were identified, some of them containing tryptophan residues (Table 1). Figure 4 shows representative fragment spectra for the peptide SVVIAYEPIWAIGTGK identified in a protein lysate from *S. aureus* in the presence of L-tryptophan (A) or 1-L-MT (B). The spectra appear almost identical in both samples, and the tryptophan-identifying fragment ions are clearly present. This example shows that proteins from bacteria grown in the presence of 1-L-MT as the only possible tryptophan-source contain normal tryptophan and not 1-L-MT or any other tryptophan modification. Overall, several tryptophan-containing peptides could be identified in all samples of bacterial proteins and they all contained tryptophan and not 1-L-MT regardless if L-Trp or 1-L-MT was added to the cell culture medium (Table 1). All identified tryptophan-containing peptides are listed in Suppl. table S1.

**Table 1:** Peptides identified in bacteria with LC-MS/MS.

bacteria	substrate	number of peptides	number of trp peptides	number of 1-L-MT peptides
*S. aureus*	L-tryptophan	390	8	0
*S. aureus*	1-L-MT	45	11	0
*S. agalactiae*	L-tryptophan	247	7	0
*S. agalactiae*	1-L-MT	300	12	0

Total numbers and numbers of tryptophan (trp) containing peptides are given for protein samples from different bacteria grown either with L-tryptophan or 1-L-MT as unique available tryptophan source. For all analyses, tryptophan methylation was used as a variable modification, but none of the peptides identified contained methylated tryptophan.

### Tryptophan contamination in 1-L-MT has an impact on IDO activity and IDO-mediated effects *in vitro*

**Figure 5:**
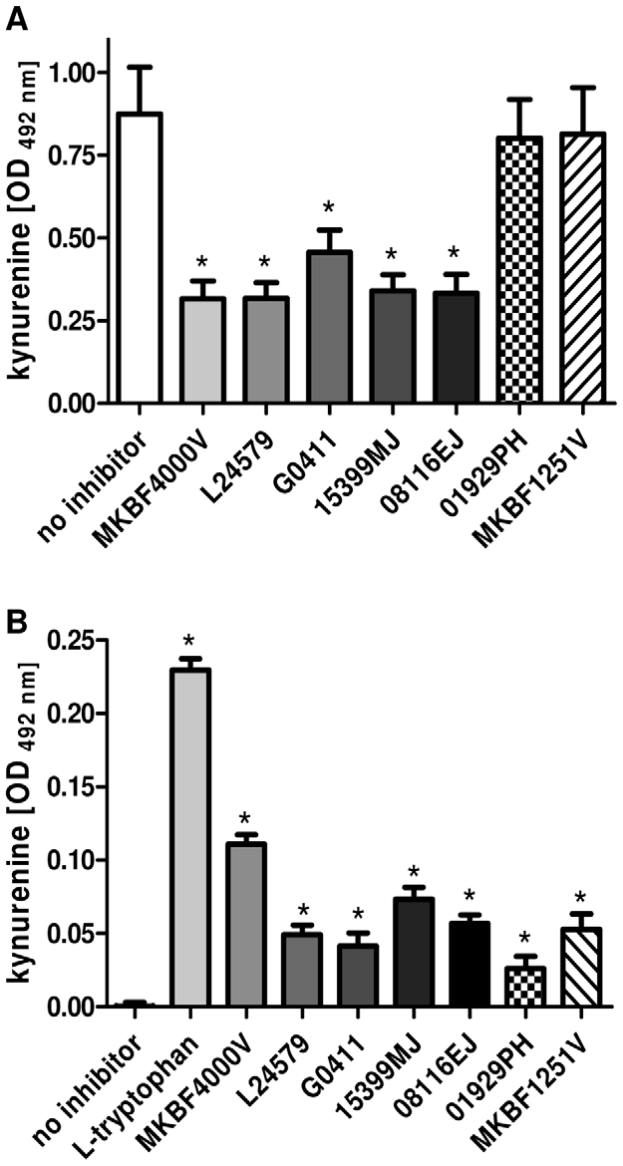
Impact of tryptophan contamination in 1-L-MT on kynurenine detection and determination of IDO activity. (A) Measurement of kynurenine in the supernatant of IFN-γ-stimulated (1000 U/mL) 86HG39 glioblastoma cells cultured in IMDM medium with additional 100 µg/mL L-tryptophan for 72 h. During this stimulation period, the cells were treated with different 1-L-MT lots (grey bars) or 1-D-MT lots (shaded bars) (200 µg/mL each). (B) Kynurenine production within different 1-L-MT lots. 86HG39 cells were cultured in custom-made, tryptophan-free cell culture medium and stimulated with IFN-γ (1000 U/mL) for 72 h in the presence of 1-MT or L-trp (200 µg/mL). In both experimental groups the kynurenine content in the cell culture supernatants was determined by optical density at 492 nm +/− SEM, using Ehrlich's reagent. A significant inhibition of kynurenine production (A) and a significant increase in kynurenine production (B) (p<0.05) as compared to the negative control is marked with an asterisk (*) n = 3.

As shown in figure 1 we have observed an altered growth of bacteria in *in vitro* experiments when we used 1-L-MT as IDO inhibitor. Therefore, it was possible that the tryptophan within 1-L-MT lots would skew the results of other *in vitro* experiments. To investigate this, we first analyzed whether the tryptophan source in 1-L-MT has any impact on the IDO inhibitory function of 1-L-MT (Fig. 5A). Therefore, we incubated IFN-γ stimulated, 86HG39 glioblastoma cells in the presence of 1-L-MT derived from the diverse lots analyzed above (200 µg/mL each). IDO enzyme activity was detected by measurement of kynurenine in cell culture supernatants using Ehrlich's Reagent. We found that all 1-L-MT lots significantly inhibited the production of kynurenine (Fig. 5A). The addition of 1-D-MT had no inhibitory effect. However, when the 1-MT was added in a high concentration (600 µg/mL), the highly contaminated lot did not decrease the IDO-mediated production of kynurenine significantly, maybe due to a cleavage of the contaminating tryptophan (Supporting Information Figure S2).

Therefore, further experiments were performed to analyze the capacity of IDO to cleave the contaminating tryptophan in the different 1-L-MT lots itself. To this end, 86HG39 glioblastoma cells were stimulated in tryptophan-free medium in the presence or absence of 1-L-MT. As expected, we detected kynurenine in the supernatants of cells stimulated in tryptophan-free medium supplemented with 1-L-MT (Fig. 5B). Here, the amount of kynurenine present in the cell supernatants correlated with the respective amount of tryptophan contained within the 1-L-MT lots. This finding indicates that this tryptophan was converted to kynurenine, although the competitive inhibitor 1-L-MT was present.

### Parasites and human T cells grow in the presence of 1-L-MT

**Figure 6:**
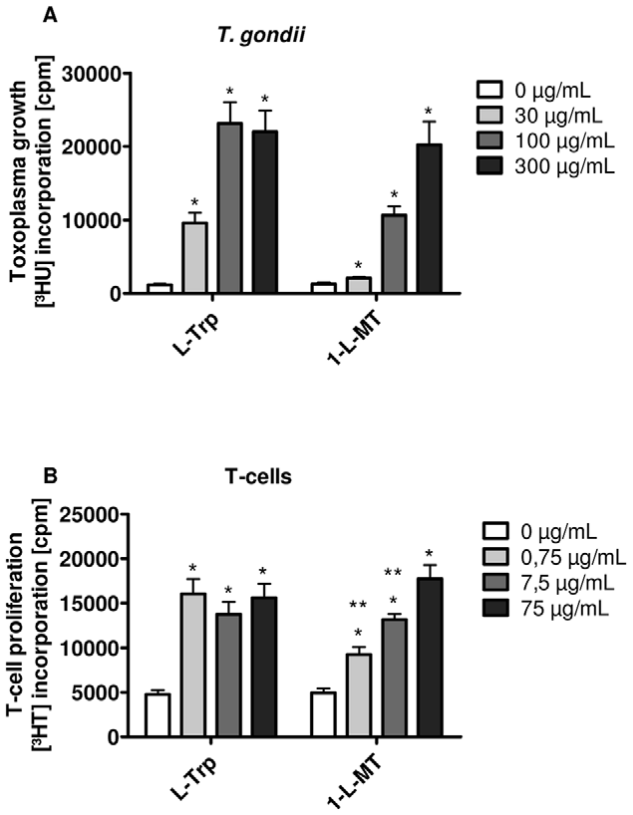
1-L-MT serves as tryptophan replacement for *Toxoplasma gondii* and human T cells. (A) *Toxoplasma gondii* growth in 86HG39 cells and (B) human T cell growth was observed in tryptophan-depleted medium in the presence of different amounts of L-tryptophan or 1-L-MT (0–300 µg/mL for *T. gondii* and 0–75 µg/mL for T cells). Parasites as well as human cells were unable to proliferate in the absence of tryptophan, but retained their ability to proliferate in the presence of L-tryptophan and, importantly, of 1-L-MT. Data are given as *T. gondii* growth, measured by [^3^H] uracil incorporation and as T-cell proliferation, measured by [^3^H] thymidine incorporation +/− SEM of three (T cells) or four (parasites) independent experiments with each experiment performed in triplicates. * = increased growth as compared to the negative control (p<0.05). ** = significant decrease as compared to the 0,75 µg/mL tryptophan group (p<0.05).

It is important to know, whether the L-tryptophan that is contained within 1-L-MT can also abrogate other IDO-mediated antimicrobial or immunoregulatory functions by directly permitting the growth of parasites or T cells. For this reason we analyzed the growth of the obligate intracellular, tryptophan-auxotroph parasite *Toxoplasma gondii* (*T. gondii*) in the presence of 1-L-MT. IFN-γ (300 U/mL) pre-stimulated 86HG39 glioblastoma cells were infected with *T. gondii* tachyzoites and defined amounts of L-tryptophan or 1-L-MT (0–300 µg/mL) were added to the culture. The parasite proliferation was then determined by [^3^H] uracil incorporation. Figure 6A clearly demonstrates that *T. gondii* was not able to grow in IFN-γ-activated 86HG39 cells as described earlier [15]. The addition of supplemental tryptophan and as well of 1-L-MT reconstituted parasite proliferation in a concentration-dependent manner. Since the maximal *T. gondii* proliferation in the presence of high amounts of tryptophan or 1-L-MT was comparable to the proliferation in conventional IMDM cell culture medium (Supporting Information Figure S3) we suggest that the tryptophan within 1-L-MT is sufficient to abrogate the IDO-mediated antiparasitic effect.

IDO-mediated immunoregulatory effects are recently of considerable scientific interest, not only in connection with autoimmune diseases, but also in the field of tumor pathology and therapy. Therefore, we tested whether the tryptophan within 1-L-MT lots allows human T cell proliferation in the absence of extra tryptophan. Like *S. aureus* and *T. gondii*, human T cells do not proliferate in tryptophan-depleted, conditioned medium harvested from IFN-γ-stimulated IDO-positive 86HG39 cells (Fig. 6B). However, the addition of either L-trp, or to a lesser extent, 1-L-MT restored the T-cell proliferation. While T cells showed a maximal proliferation after the supplementation with 0.75 µg/mL L-trp, the amount of 1-L-MT necessary to reconstitute the growth was more than 10 fold higher, corresponding to the fact that the 1-L-MT used for this experiment (lot 08116EJ) was contaminated with tryptophan. Additionally, this result also reveals that the overall T-cell growth was not affected by 1-L-MT as there was an equal rate of [^3^H] thymidine incorporation in the presence of maximal amounts of L-trp or 1-L-MT.

This incorporation of the tryptophan contained within 1-L-MT lots was also analyzed in competitive assays. In these assays T cells were cultured in conditioned, tryptophan-free cell culture medium, supplemented with radioactively labeled [^3^H] L-trp and different amounts of unlabelled L-trp or 1-L-MT. Changes in the incorporation of [^3^H] L-trp were determined via liquid scintillation spectrometry. The L-tryptophan within 1-L-MT was able to compete with natural tryptophan sources *in vitro* as expected (Supporting Information Figure S4).

**Figure 7:**
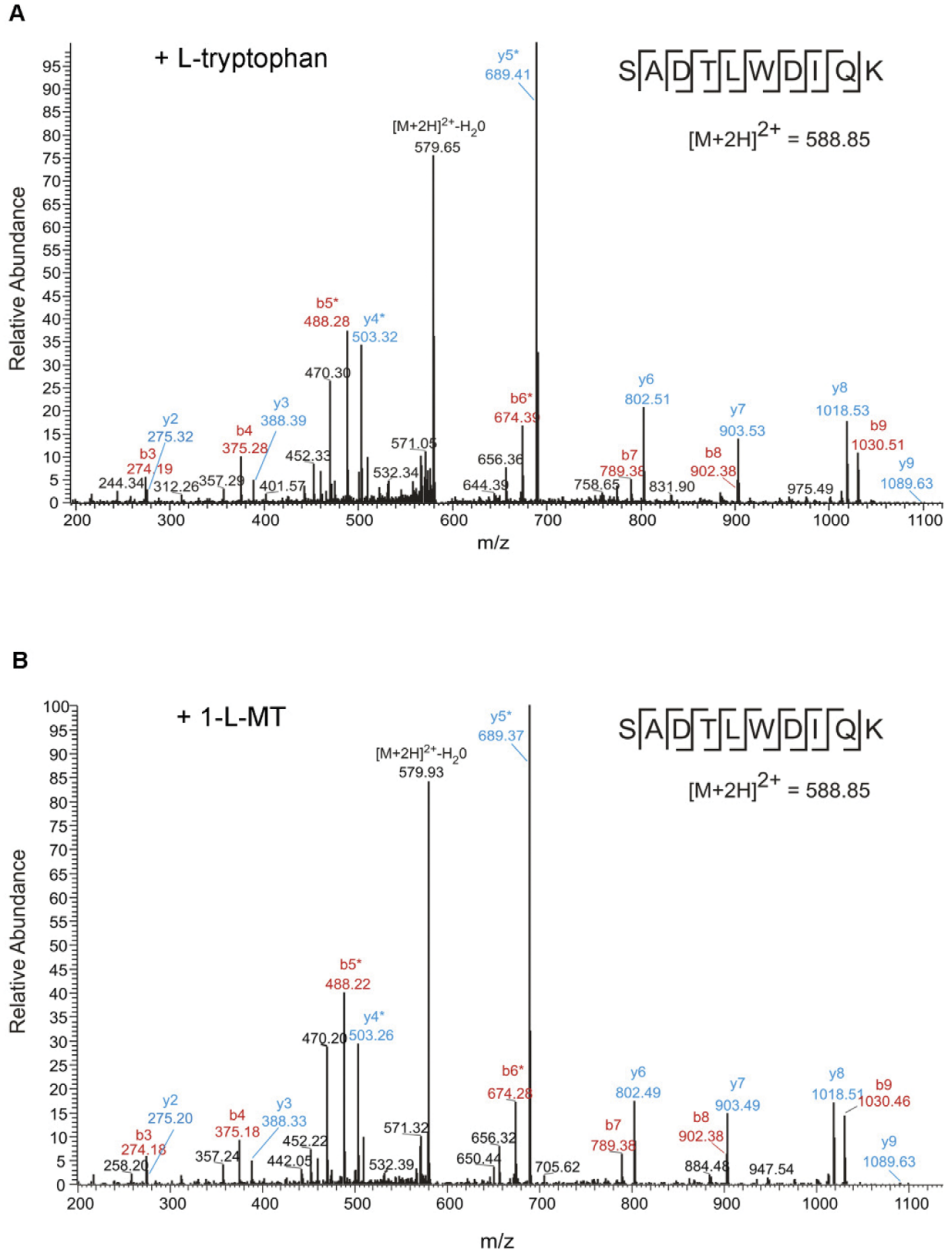
MS/MS spectra for a tryptophan-containing peptide from 86HG39 cell culture lysate. 86HG39 cells were grown in the presence of L-tryptophan (A) or 1-L-MT (B). From the spectra shown, the peptide SADTLWDIQK from human L-lactate dehydrogenase B chain was identified with a mascot score of 54 in both samples. For cells grown in the presence of L-tryptophan or 1-L-MT, the spectra appear almost identical. Fragment ions identifying the tryptophan residue are clearly visible and are marked with an asterisk.

Further analyses were carried out to investigate if the tryptophan in 1-L-MT is proteinogenic in other human cells. Therefore, two different human cell lines were cultured for 15 weeks in tryptophan-free cell culture medium, supplemented with tryptophan-free serum substitute, with additional L-trp or 1-L-MT (100 µg/mL each), respectively. Thereafter, the cells were lysed and the proteins were analyzed via LC-MS/MS. These analyses identified several tryptophan-containing peptides in both experimental groups, regardless of the tryptophan source added. The number of the identified peptides is given in table 2. Exemplary fragment spectra for the peptide SADTLWDIQK from 86HG39 cells cultivated with L-tryptophan (A) or 1-L-MT (B) are shown in figure 7. All identified tryptophan-containing peptides are listed in Suppl. table S2. None of the listed peptides contained Methyl-tryptophan instead of tryptophan.

**Table 2:** Peptides identified in human cells with LC-MS/MS.

cell line	substrate	number of peptides	number of trp peptides	number of 1-L-MT peptides
86HG39	L-tryptophan	238	9	0
86HG39	1-L-MT	245	3	0
HeLa	L-tryptophan	224	10	0
HeLa	1-L-MT	239	6	0

Total numbers and numbers for tryptophan (trp) containing peptides are given for protein samples from different human tumor cells grown either with L-tryptophan or 1-L-MT as the only available tryptophan source. For all analyses, tryptophan methylation was used as a variable modification, but none of the identified peptides contained methylated tryptophan.

Together, we have shown that the addition of 1-L-MT is able to reverse IDO-mediated effects in *in vitro* experiments. It allowed the growth of both *T. gondii* and human T cells. This was proven to be due to contaminating L-tryptophan within all commercially available 1-L-MT lots tested. Our data underline that in our *in vitro* conditions the contaminating L-trp can reverse the IDO inhibition of 1-L-MT independently of its function as IDO inhibitor.

## Discussion

For the last two decades 1-Methyl-tryptophan (1-MT) has been the most frequently used inhibitor of the tryptophan-degrading enzyme indoleamine 2,3-dioxygenase (IDO). However, whether 1-D-MT or 1-L-MT is more effective has remained unanswered. It has been shown that the L isomer is efficient in inhibiting IDO activity in different human cell lines and tumor cells [13] and in cell-free systems [11]. Hence, 1-L-MT seems to be the prominent IDO inhibitor in *in vitro* assays, but it is less active in *in vivo* systems. 1-D-MT is also a potent IDO inhibitor and is able to reverse the immunoregulatory function of IDO-expressing human and murine antigen-presenting cells *in vitro* and it serves as antitumor agent in murine *in vivo* tumor models [16]. Additionally, 1-D-MT has been shown to abrogate the IDO-mediated inhibition of *Toxoplasma gondii* in *in vivo* infections of mice [18].

The discrepancies between the results obtained using 1-L-MT or 1-D-MT have not been fully elucidated yet, although there have been several explanatory approaches. One is that 1-D-MT inhibits not only IDO, but also IDO-2 and that IDO-2 might therefore account for the effects observed [17]. In contrast some results show that 1-L-MT, not 1-D-MT, is the more effective inhibitor of vertebrate IDO-2 enzyme [19]. Other examples of such IDO-independent effector mechanisms of 1-MT are the fact that 1-DL-MT, the racemic form, interferes with TLR signaling in dendritic cells [20] and that it not only inhibits IDO enzyme activity, but also IDO expression at a transcriptional and translational level [21], [22]. Because of this, the expression of other proteins might also be impaired due to unspecific side-effects mediated by 1-MT.

In this manuscript we offer an additional explanation for the conflicting results using the different 1-MT isoforms. 1-L-MT, but not 1-D-MT, contains L-tryptophan that is proteinogenic for human cells as well as for tryptophan-auxotroph bacteria and parasites. When the cells or the microorganisms are cultivated *in vitro* in the absence of L-tryptophan, but in the presence of 1-L-MT, they are able to proliferate normally. This clearly demonstrates that 1-L-MT facilitates the growth of human cells and microorganisms independently of previous IDO-mediated tryptophan depletion. However, this growth of microorganisms or T cells may also be the result of a successful inhibition of IDO activity *in vitro* and has been interpreted as a 1-L-MT mediated inhibition of IDO [13], [23], [24]. To date, a potential contaminations of 1-MT with tryptophan has not been taken into account but must be considered in retrospective interpretation of these data.

Although 1-D-MT contains most likely D-tryptophan that has no proteinogenic potential, commercially available 1-L-MT is contaminated with proteinogenic L-tryptophan. This is an obvious functional difference between both 1-MT isoforms that might in part explain the functional differences of 1-L-MT and 1-D-MT in *in vitro* and *in vivo* experiments.

Nevertheless, these results cannot fully account for the observation that 1-D-MT has strong antitumor capacities *in vivo*. In fact, 1-D-MT is a poor IDO inhibitor *in vitro*, shown in this manuscript and in others [13] and its antitumor function could be the result of different scenarios. One is that the 1-D-MT administered could still reach a tumor in concentrations sufficient for an effective inhibition of IDO. On the other hand additional, isomer-specific factors may account for the antitumoral 1-D-MT. In this context it may be of interest that IDO can act stereo-unspecifically, cleaving L-tryptophan as well as D-tryptophan [25] and that 1-L-MT is a slow substrate, at least for human IDO [26]. It would be interesting to know, whether IDO can also cleave 1-D-MT, maybe resulting in products that could alter other stereo-selective pathways.

The tryptophan contamination in 1-L-MT constitutes up to 7 µg/mg 1-MT, depending on the lot tested. Interestingly, about 200 µg/mL 1-L-MT have been effective in a number of *in vitro* experiments published. The tryptophan amount within this 1-L-MT supplementation is definitively sufficient to allow the maximal growth of cells and microorganisms that require less than 1 µg/mL for normal growth. Therefore a given T-cell proliferation must be reconsidered in the light of whether the results were due to an inhibition of IDO or if the tryptophan contained within the 1-L-MT lot permitted the growth directly.

IDO binds tryptophan with a high affinity (K_m_ = 20 µM), whereas 1-MT is a competitive inhibitor with a K_i_ of 34.4 µM [27]. Therefore, the competition of both molecules at the catalytic site of IDO favors the degradation of tryptophan, and tryptophan contaminations of 1-MT become important. With respect to the production of kynurenine not only the degree of contamination of the 1-MT lots itself, but also the concentration of 1-MT used in the experiments is crucial. However, if 1-MT is used in the standard concentration (200 µg/mL) all analyzed 1-MT lots inhibited IDO comparably.

Our findings show that a more detailed analysis of the *in vitro* experiments performed so far is necessary and may help to understand the discrepancies that occur when the two 1-MT isoforms are used *in vitro* and *in vivo*. Our data highlight the need for improved purification methods to obtain 1-L-MT devoid of L-trp contaminations in order to allow the investigation of effects mediated by 1-L-MT alone. The understanding of 1-MT mediated IDO inhibition is particularly important with regard to a pharmacologic inhibition of IDO *in vitro*. *In vivo* a tryptophan contamination of 1-MT has presumably no effects since excessive amounts of L-tryptophan will be degraded by tryptophan 2,3-dioxygenase which is responsible for tryptophan homoeostasis in general. For *in vivo* experimentations a knock down of IDO gene expression in addition to pharmacological inhibition should be taken into account.

## Materials and Methods

### Cell lines and culture

HeLa [15] and glioblastoma cells 86HG39 [28] were cultured in Isocove's Modified Dulbecco's Medium (IMDM) containing 5% fetal calf serum (FCS, Cambrex, East Rutherford) in culture flasks (Costar, Cambridge). Cultures were split every 4 days into 1∶10 ratios using 0.25% trypsin-EDTA (Gibco, Grand Island).

Peripheral blood mononuclear cells (PBMC) were prepared from heparinised blood of healthy donors after density gradient centrifugation.

*Toxoplasma gondii* tachyzoites (RH strain, ATCC, LGC Standards, Wesel, Germany) were maintained in HFF (ATCC, LGC Standards, Wesel, Germany) or glioblastoma [28] cells in IMDM containing 5% FCS. Tachyzoites were usually harvested after 3 or 5 days of incubation, resuspended in PBS and used for infection experiments.

For indicated experiments bacteria and human cells were grown in custom-made, tryptophan-free Roswell Park Memorial Institute (RPMI 1640) medium (PAN Biotech, Aidenbach, Germany) containing 0,3 mg/mL L-glutamine and 10% custom-made, tryptophan-free serum-substitute Panexin NTA (PAN Biotech, Aidenbach, Germany). This medium was free of tryptophan as determined by HPLC analysis. For growth experiments additional 100 µg/mL L-tryptophan or 1-L-MT were supplemented.

### Ethics statement

This study obtained ethics approval from the ethics committee of the Medical Faculty of the Heinrich-Heine University Düsseldorf (study no. 3838). Human PBMC were generated from the blood of healthy individuals after informed and written consent.

### Reagents and tryptophan analogues

Recombinant human IFN-γ was purchased from Tebu-Bio (Offenbach, Germany).

### Table 3: Sources of used substances

**Table 3:** Sources of used substances.

substance	LOT	denoted purity [%]	company
L-tryptophan	113H0290	98*	Sigma-Aldrich (St. Louis, USA)
1-Methyl-L-tryptophan	15399MJ	95*	Sigma-Aldrich (St. Louis, USA)
1-Methyl-L-tryptophan	08116EJ	95*	Sigma-Aldrich (St. Louis, USA)
1-Methyl-L-tryptophan	MKBF4000V	95*	Sigma-Aldrich (St. Louis, USA)
1-Methyl-L-tryptophan	L24579	95	Enzo Life Sciences Inc. (Farmingdale, USA)
1-Methyl-DL-tryptophan	G0411	98	Santa Cruz Biotechnology Inc. (Santa Cruz, USA)
1-Methyl-D-tryptophan	01929PH	95*	Sigma-Aldrich (St. Louis, USA)
1-Methyl-D-tryptophan	MKBF1251V	95*	Sigma-Aldrich (St. Louis, USA)

* based on elemental analysis.

1-L-MT and 1-D-MT were dissolved in 1 M NaOH to a concentration of 1 M and afterwards diluted with tryptophan-free RPMI medium to a 4 mg/mL stock solution. Experiments were performed with Sigma-Aldrich lot 08116EJ if not itemised elsewhere.

L-tryptophan was dissolved in tryptophan-free RPMI medium to a 4 mg/mL stock solution. [5-^3^H] L-tryptophan was purchased from Hartmann Analytic GmbH (Braunschweig, Germany).

### Stimulation of HeLa cells and preparation of conditioned media

HeLa cells were treated for 72 h with 500 U/mL IFN-γ to induce IDO expression [15]. Thereafter, the tryptophan and kynurenine amount in the supernatant of the cells was determined in order to test the IDO-mediated degradation of tryptophan. Afterwards, tryptophan-free, conditioned media were used as culture medium for bacteria, *Toxoplasma gondii* or human cells [15].

### Kynurenine measurement

The kynurenine content in the supernatants of the cells was determined photometrically as described before using Ehrlich's reagent [29] or by HPLC.

### HPLC analysis

1-MT and L-tryptophan were dissolved in custom-made, tryptophan-free cell culture medium (4 mg/mL stock). 200 µl samples were then diluted with 200 µl PBS and 400 µl internal control (3-nitrotyrosine, 50 µM, Sigma Aldrich, St. Louis, USA). Thereafter, proteins within the samples were precipitated with 1/9 (v/v) 30% trichloroacetic acid. After centrifugation, the supernatant was analyzed. The tryptophan, 1-MT and kynurenine amount was quantified, using the Gold-Universal-Grad System (BeckmanCoulter, Krefeld, Germany), containing a 126 solvent module and the 166 detector, together with a 3 µm reversed phase column (RP-C18endcapped, Merck, Darmstadt, Germany). The separation was performed in sodium acetate buffer (50 mM/pH 4.2) with an increasing gradient of acetonitrile, using a flow rate of 1.0 mL/min. The absorbance of the column effluent was monitored at 280 nm. All peaks were identified by means of comparison with the retention time of standards, which was previously determined.

### Determination of bacterial growth

Conditioned media or custom-made, tryptophan-free RPMI 1640 medium containing 0,3 mg/mL L-glutamine and 10% custom-made, tryptophan-free serum-substitute Panexin NTA were infected with *Staphylococcus aureus* (*S. aureus*) derived from our routine diagnostic and supplemented with different amounts of L-trp or 1-L-MT (0–200 µg/mL). IDO sensitivity of these strains was analyzed previously [30], [31]. For infection experiments, a single bacterial colony was suspended in 5 mL PBS. Bacteria were serially diluted in PBS and 10 µL were added to 200 µl of conditioned medium, corresponding to 10–100 CFU (colony forming units) per well. Bacterial growth was monitored after an incubation of 16 h with a microplate photometer (SLT Labinstruments, Crailsheim, Germany) measuring the optical density at 620 nm. As a control, bacteria were grown on brain heart infusion agar (Difco, Hamburg, Germany) containing 5% sheep blood, incubated at 37°C in 5% CO_2_-enriched atmosphere.

For long-term experiments *S. aureus* was cultured in tryptophan-free, conditioned medium in the absence or in the presence of 1-L-MT. The supernatant containing the bacteria was diluted daily in PBS and afterwards used to infect fresh media. Each day the bacterial growth was determined by measuring of the optical density at 620 nm.

In addition all other bacteria (*Streptococcus agalactiae, Enterococcus faecalis, Pseudomonas aeruginosa, Klebsiella pneumoniae, Echerichia coli, Proteus mirabilis, Serratia marcescens, Salmonella enteridis*) were also derived from clinical specimens and used for experiments as described above.

### *Toxoplasma gondii* proliferation assay

3×10^4^ gliobastoma 86HG39 cells/well were incubated in 96 well plates and treated with IFN-γ(300 U/mL) or left unstimulated. After 72 h 3×10^4^ RH strain *Toxoplasma gondii* tachyzoites were added to the cells and L-trp or 1-L-MT were supplemented (0; 10; 30 or 100 µg/mL). After additional 48 h 0.33 µCi [5,6-^3^H] uracil/well was added and the uracil incorporation into the *T. gondii* was determined 24 h later by freeze/thaw lysis of the cells and subsequent analysis using liquid scintillation spectrometry (1205 Betaplate, PerkinElmer, Jugesheim, Germany).

### T cell proliferation in the presence of 1-L-MT

Two different types of T-cell experiments were carried out. First of all, T-cell growth was determined via the [^3^H] thymidine incorporation assay. For this 1.5×10^5^ peripheral blood lymphocytes were incubated in tryptophan-free conditioned medium supplemented with different amounts of L-trp or 1-L-MT (0; 0.75; 7.5 or 75 µg/mL). After 72 h T-cell proliferation was determined by adding 0.2 µCi [^3^H] thymidine for 24 h and using liquid scintillation spectrometry (1205 Betaplate, PerkinElmer, Jugesheim, Germany).

The second assay was based on the replacement of 0.75 µg/mL radioactively labelled [5-^3^H] L-tryptophan ([^3^H] L-trp) in the protein fraction by different amounts of unlabelled L-trp or 1-L-MT (0; 0.75; 7.5 or 75 µg/mL). Here, 1.5×10^5^ peripheral blood lymphocytes were incubated in tryptophan-free, conditioned medium supplemented with [^3^H] L-trp and the different substrates of interest. After 72 h the tryptophan incorporation into the T cells was determined using liquid scintillation spectrometry. [^3^H] L-trp incorporation into the cells was analyzed after several washing steps, freeze-and-thaw cycles and precipitation with 30% trichloroacetic acid. The T cell proliferation was driven in both assays by the addition of the monoclonal anti-CD3 antibody OKT3 (American Type Culture Collection, Rockville) to freshly isolated human peripheral lymphocytes [15].

### Cultivation of human cells and bacteria for liquid chromatography and tandem mass spectrometry (LC-MS/MS) analysis

The human glioblastoma cell line 86HG39 and HeLa cells were cultured in tryptophan-free RPMI 1640 cell culture medium supplemented with 10% tryptophan-free Panexin NTA (both PAN Biotech, Germany) for 15 weeks. Cells were split every 4 days into 1∶10 ratios using 0.25% trypsin-EDTA (Gibco, Grand Island).

*Staphylococcus aureus* and *Streptococcus agalactiae* were incubated in RPMI 1640 cell culture medium supplemented with 10% tryptophan-free Panexin NTA for 18 h. Thereafter, cells were lysed by freeze-thaw cycles in PBS containing a protease inhibitor cocktail (Roche Diagnostics GmbH, Germany).

### Gel electrophoresis and tryptic digestion of proteins

For sample clean-up, proteins were shortly run on a 4–12% NuPageTM Bis-Tris gradient gel (Invitrogen) according to the manufacturer's instructions and stained with colloidal Coomassie Brilliant Blue G-250. Each sample was cut out as one band and immediately destained, washed, subjected to tryptic digestion, and prepared for LC/MS analysis as described previously [32].

### Liquid chromatography tandem mass spectrometry (LC-MS/MS) analysis

Peptides were separated using the Ultimate 3000 RSLCnano HPLC system (Dionex, now Thermo Fisher Scientific, Idstein, Germany). For human cell culture samples, the HPLC was coupled to an LTQ Velos instrument (Thermo Fisher Scientific, Bremen, Germany), while bacterial samples were analysed using an LTQ Orbitrap Velos instrument (Thermo Fisher Scientific, Bremen, Germany).

For peptide separation, a C18 RP nano LC column (75 µm inner diameter, 250 mm, particle size 2 µm PepMap, Dionex LC Packings) and a binary solvent system consisting of 0.1% (v/v) formic acid (solvent A) and 0.1% (v/v) formic acid in 84% (v/v) acetonitrile (solvent B) with the following linear gradient were used: 5–40% solvent B in 35 min and 40–95% solvent B in 5 min. Columns were operated at a constant temperature of 60°C. The LC was coupled to the respective mass spectrometer using a nano-electrospray ion source (Thermo Fisher Scientific) and distal coated Silica Tips (FS360-20-10-D, New Objective, Woburn, MA).

For the LTQ Velos measurements, full MS spectra in enhanced modus were recorded for m/z 300–1500. The five most intense ions were selected for data-dependent fragmentation (MS/MS) by low energy collision-induced dissociation in the linear ion trap.

For LTQ Orbitrap Velos analysis, survey scans (300–2000 m/z) were recorded with a resolution of 30000 for m/z = 400. A data-dependent fragmentation was carried out for the 20 most intense ions, and fragment ions were analysed in the linear ion trap. For both instruments, dynamic exclusion was used to prevent fragmentation of previously selected precursors.

Raw files were processed using ProteomeDiscoverer 1.2 (Thermo Fisher Scientific). MS/MS spectra were searched using the Mascot search engine against the following databases: Swissprot (SwissProt 57.15, Taxonomy: Homo sapiens (human), 20,266 sequences) for human cell culture samples and UniProt (Taxonomy: Bacteria (Eubacteria), 323,530 sequences) for bacterial samples. The search was carried out with the following settings: precursor and fragment mass tolerance 0.4 Da for LTQ Velos data and 5 ppm/0.4 Da for LTQ Orbitrap data, one missed cleavage, oxidation of methionine and methylation of tryptophan as variable modifications. Peptide identifications were filtered at 1% false discovery rates (FDR). Protein grouping was enabled and only rank 1 peptides were considered, additional peptides with the same score were excluded.

### Matrix Assisted Laser Desorption Ionization Mass Spectrometry (MALDI-MS) Analysis

For the MALDI-MS analysis different 1-L-Methyl-tryptophan lots were dissolved in water in a concentration of 1 µg/µl. The measurements were performed using a MALDI-TOF instrument Voyager-DE-STR (Applied Biosystem, now AB Sciex, Framingham, Massachusetts; USA) with a nitrogen laser (λ = 337 nm) operating in reflector mode with 25 kV acceleration voltage. The samples were prepared by the standard dried droplet method, by mixing 0.5 µl of sample solution with 2 µl of 2,5-dihydroxybenzoic acid (DHB) matrix solution (10 mg in 1 ml water) on target. The droplet was dried by a gentle flow of air. An internal calibration was done with the monomer ions of the matrix. The spectra were obtained by averaging 100 laser shots.

### Electrospray Ionization tandem mass spectrometry (ESI-MS/MS) analysis

The ESI-MS/MS measurements were performed with an ESI-QqTOF hybrid tandem mass spectrometer QSTAR XL (Applied Biosystems, now AB Sciex, Framingham, Massachusetts; USA) equipped with a nanospray ion source.

### Statistical analysis

All cell culture experiments were done in triplicates and data are given as mean +/− SEM of a minimum of three independent experiments, if not itemized elsewhere. For statistical analysis the unpaired t-test was used and p values<0.05 were considered significant. The analysis was performed with GraphPad Prism software (La Jolla, California, USA).

## Supporting Information

Figure S1 **MS Analysis of different 1-L-MT lots dissolved in tryptophan-free cell culture medium.** Exemplary spectra of the lots (A) MKBF4000V, (B) 08116EJ, (C) 15399MJ (all derived from Sigma Aldrich, St. Louis, USA) and (D) G0411 (derived from Santa Cruz Biotechnology Inc., Santa Cruz, USA). Additional to 1-L-MT with the m/z of 219, all lots also contain tryptophan with the m/z of 205.
(PDF)

Figure S2 **Impact of tryptophan contamination in 1-L-MT on IDO inhibitory function.** Measurement of kynurenine in the supernatant of IFN-γ stimulated (1000 U/mL) or unstimulated 86HG39 glioblastoma cells cultured in IMDM medium with additional 100 µg/mL L-tryptophan for 72 h. During this stimulation period, the cells were treated with different 1-L-MT or 1-D-MT lots (600 µg/mL each). The kynurenine content in the cell culture supernatants was determined by optical density at 492 nm +/− SEM, using Ehrlich's reagent. A significant inhibition of kynurenine production (p<0.05) as compared to the negative control is marked with an asterisk (*) n = 3.
(PDF)

Figure S3 ***Toxoplasma gondii*** **proliferation in IMDM medium with additional Ltryptophan or 1-L-MT.** The proliferation of *Toxoplasma gondii* in tryptophan-containing IMDM medium with additional L-tryptophan or 1-L-MT (100 µg/mL each) was determined. The supplementation of the substrates had no negative effect on parasite growth. Data are given as [3H] uracil incorporation +/− SEM of four independent experiments, each performed in triplicates.
(PDF)

Figure S4 **Competitive replacement of [3H] L-tryptophan by unlabelled L-tryptophan and 1-L-MT in human T cells.** OKT3-stimulated T cells were cultured in conditioned, tryptophan-free cell culture medium containing 0,75 µg/mL [3H] L-tryptophan and additional L-tryptophan and 1-L-MT (0–75 µg/mL each). The [3H] L-tryptophan was detected by liquid scintillation spectrometry. Its incorporation into T cells was significantly decreased in a concentration-dependent way, when it was competitively replaced by unlabelled L-tryptophan and 1-L-MT. Data are given as [3H]-tryptophan incorporation, measured by liquid scintillation spectrometry, +/− SEM of four independent experiments with each experiment performed in triplicates.
(PDF)

Table S1 **Tryptophan-containing peptides identified by mass spectrometry.** Protein extracts from bacteria were analyzed by LC-MS. Mascot-Scores for the identified tryptophan-containing peptides are given for bacteria grown in the presence of L-Tryptophan (Trp) and bacteria grown in the presence of 1-L-MT. n.i.: not identified. All searches were carried out with methylation of tryptophan residues as variable modifications. No peptide with tryptophan methylation was identified in any sample.
(PDF)

Table S2 **Tryptophan-containing peptides identified by mass spectrometry.** Protein extracts from human cells were analysed by LC-MS. Mascot-Scores for the identified tryptophan-containing peptides are given for cells grown in the presence of LTryptophan (Trp) and cells grown in the presence of 1-L-MT. n.i.: not identified. All searches were carried out with methylation of tryptophan residues as variable modifications. No peptide with tryptophan methylation was identified in any sample.
(PDF)
