# Supplementary material for: Influence of Tryptophan Contained in 1-Methyl-Tryptophan on Antimicrobial and Immunoregulatory Functions of Indoleamine 2,3-Dioxygenase
Source: PLoS One. 2012 Sep 13;7(9):e44797. doi: 10.1371/journal.pone.0044797 (PMC3441469; doi:10.1371/journal.pone.0044797)
Supplement: Figure S1 — MS Analysis of different 1-L-MT lots dissolved in tryptophan-free cell culture medium. Exemplary spectra of the lots (A) MKBF4000V, (B) 08116EJ, (C) 15399MJ (all derived from Sigma Aldrich, St. Louis, USA) and (D) G0411 (derived from Santa Cruz Biotechnology Inc., Santa Cruz, USA). Additional to 1-L-MT with the m/z of 219, all lots also contain tryptophan with the m/z of 205. (PDF) [file pone.0044797.s001.pdf]

Supplemental figure S1

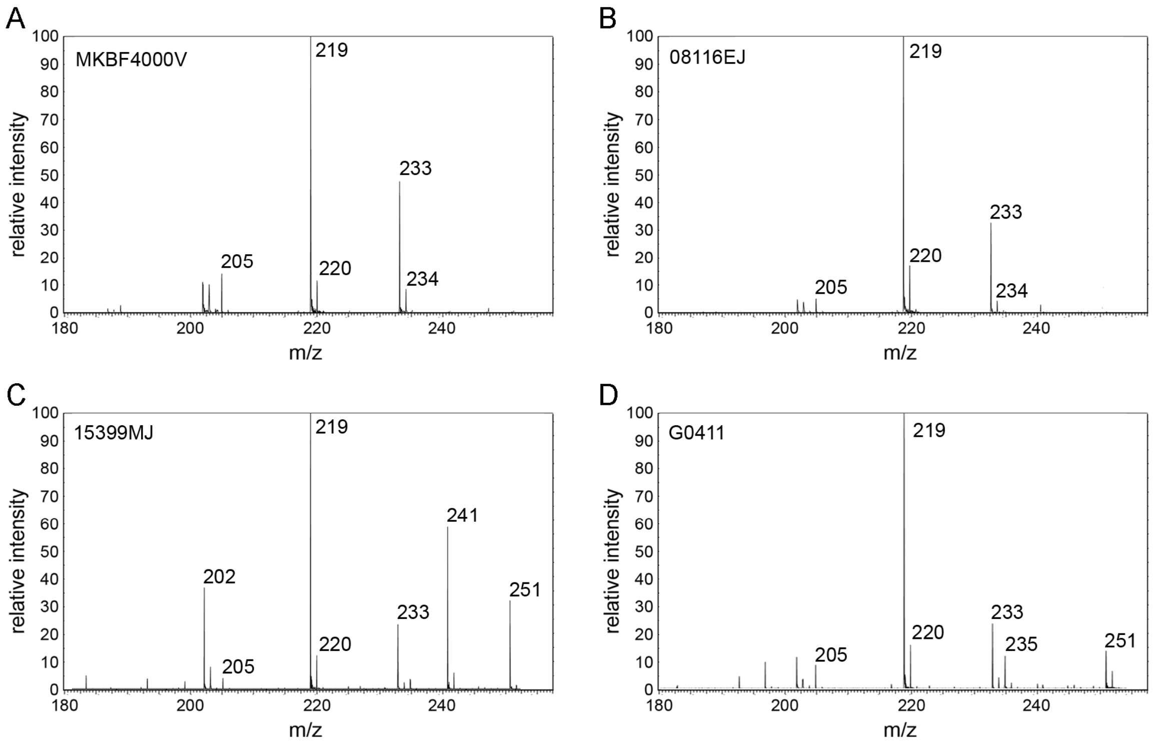

Figure S1. MS Analysis of different 1-L-MT lots dissolved in tryptophan-free cell culture medium. Exemplary spectra of the lots (A) MKBF4000V, (B) 08116EJ, (C) 15399MJ (all derived from Sigma Aldrich, St. Louis, USA) and (D) G0411 (derived from Santa Cruz Biotechnology Inc., Santa Cruz, USA). Additional to 1-L-MT with the m/z of 219, all lots also contain tryptophan with the m/z of 205.
