# Supplementary material for: Influence of Tryptophan Contained in 1-Methyl-Tryptophan on Antimicrobial and Immunoregulatory Functions of Indoleamine 2,3-Dioxygenase
Source: PLoS One. 2012 Sep 13;7(9):e44797. doi: 10.1371/journal.pone.0044797 (PMC3441469; doi:10.1371/journal.pone.0044797)
Supplement: Figure S2 — Impact of tryptophan contamination in 1-L-MT on IDO inhibitory function. Measurement of kynurenine in the supernatant of IFN-γ stimulated (1000 U/mL) or unstimulated 86HG39 glioblastoma cells cultured in IMDM medium with additional 100 µg/mL L-tryptophan for 72 h. During this stimulation period, the cells were treated with different 1-L-MT or 1-D-MT lots (600 µg/mL each). The kynurenine content in the cell culture supernatants was determined by optical density at 492 nm +/− SEM, using Ehrlich's reagent. A significant inhibition of kynurenine production (p<0.05) as compared to the negative control is marked with an asterisk (*) n = 3. (PDF) [file pone.0044797.s002.pdf]

Supplemental figure S2

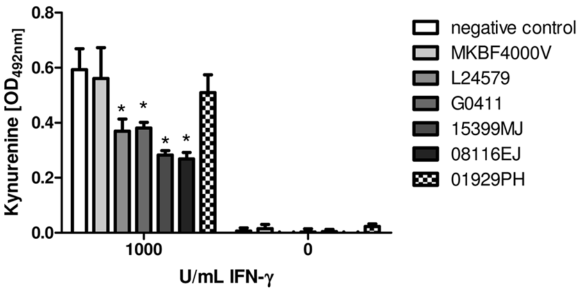

Figure S2. Impact of tryptophan contamination in 1-L-MT on IDO inhibitory function. Measurement of kynurenine in the supernatant of IFN-γ–stimulated (1000 U/mL) or unstimulated 86HG39 glioblastoma cells cultured in IMDM medium with additional 100 µg/mL L-tryptophan for 72 h. During this stimulation period, the cells were treated with different 1-L-MT or 1-D-MT lots (600 µg/mL each). The kynurenine content in the cell culture supernatants was determined by optical density at 492 nm +/- SEM, using Ehrlich´s reagent. A significant inhibition of kynurenine production ($p < 0.05$) as compared to the negative control is marked with an asterisk (*) n = 3.
