# Supplementary material for: Influence of Tryptophan Contained in 1-Methyl-Tryptophan on Antimicrobial and Immunoregulatory Functions of Indoleamine 2,3-Dioxygenase
Source: PLoS One. 2012 Sep 13;7(9):e44797. doi: 10.1371/journal.pone.0044797 (PMC3441469; doi:10.1371/journal.pone.0044797)
Supplement: Figure S3 — Toxoplasma gondii proliferation in IMDM medium with additional Ltryptophan or 1-L-MT. The proliferation of Toxoplasma gondii in tryptophan-containing IMDM medium with additional L-tryptophan or 1-L-MT (100 µg/mL each) was determined. The supplementation of the substrates had no negative effect on parasite growth. Data are given as [3H] uracil incorporation +/− SEM of four independent experiments, each performed in triplicates. (PDF) [file pone.0044797.s003.pdf]

Supplemental figure S3

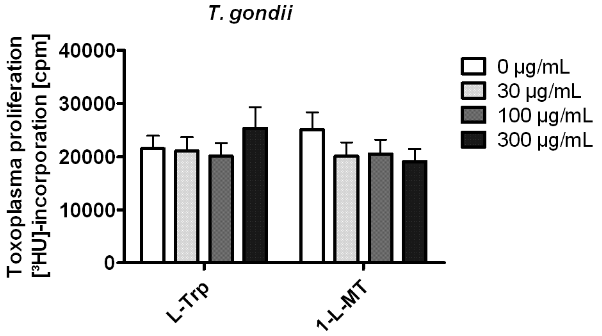

Figure S3. *Toxoplasma gondii* proliferation in IMDM medium with additional L-tryptophan or 1-L-MT. The proliferation of *Toxoplasma gondii* in tryptophan-containing IMDM medium with additional L-tryptophan or 1-L-MT (100 µg/mL each) was determined. The supplementation of the substrates had no negative effect on parasite growth. Data are given as [^{3}H] uracil incorporation +/- SEM of four independent experiments, each performed in triplicates.
