# Supplementary material for: Influence of Tryptophan Contained in 1-Methyl-Tryptophan on Antimicrobial and Immunoregulatory Functions of Indoleamine 2,3-Dioxygenase
Source: PLoS One. 2012 Sep 13;7(9):e44797. doi: 10.1371/journal.pone.0044797 (PMC3441469; doi:10.1371/journal.pone.0044797)
Supplement: Figure S4 — Competitive replacement of [3H] L-tryptophan by unlabelled L-tryptophan and 1-L-MT in human T cells. OKT3-stimulated T cells were cultured in conditioned, tryptophan-free cell culture medium containing 0,75 µg/mL [3H] L-tryptophan and additional L-tryptophan and 1-L-MT (0–75 µg/mL each). The [3H] L-tryptophan was detected by liquid scintillation spectrometry. Its incorporation into T cells was significantly decreased in a concentration-dependent way, when it was competitively replaced by unlabelled L-tryptophan and 1-L-MT. Data are given as [3H]-tryptophan incorporation, measured by liquid scintillation spectrometry, +/− SEM of four independent experiments with each experiment performed in triplicates. (PDF) [file pone.0044797.s004.pdf]

Supplemental figure S4

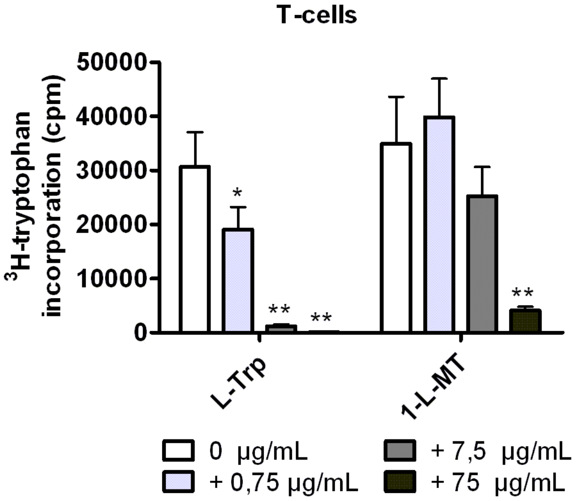

Figure S4. Competitive replacement of [^{3}H] L-tryptophan by unlabelled L-tryptophan and 1-L-MT in human T cells. OKT3-stimulated T cells were cultured in conditioned, tryptophan-free cell culture medium containing 0,75 µg/mL [^{3}H] L-tryptophan and additional L-tryptophan and 1-L-MT (0-75 µg/mL each). The [^{3}H] L-tryptophan was detected by liquid scintillation spectrometry. Its incorporation into T cells was significantly decreased in a concentration-dependent way, when it was competitively replaced by unlabelled L-tryptophan and 1-L-MT. Data are given as [^{3}H]-tryptophan incorporation, measured by liquid scintillation spectrometry, +/- SEM of four independent experiments with each experiment performed in triplicates.
