# Supplementary material for: Influence of Tryptophan Contained in 1-Methyl-Tryptophan on Antimicrobial and Immunoregulatory Functions of Indoleamine 2,3-Dioxygenase
Source: PLoS One. 2012 Sep 13;7(9):e44797. doi: 10.1371/journal.pone.0044797 (PMC3441469; doi:10.1371/journal.pone.0044797)
Supplement: Table S1 — Tryptophan-containing peptides identified by mass spectrometry. Protein extracts from bacteria were analyzed by LC-MS. Mascot-Scores for the identified tryptophan-containing peptides are given for bacteria grown in the presence of L-Tryptophan (Trp) and bacteria grown in the presence of 1-L-MT. n.i.: not identified. All searches were carried out with methylation of tryptophan residues as variable modifications. No peptide with tryptophan methylation was identified in any sample. (PDF) [file pone.0044797.s005.pdf]

Supplemental table S1. Tryptophan-containing peptides identified by mass spectrometry.

			Trp	1-L-MT
**Sequence**	**Protein accession**	**protein description**	**Mascot Score**	**Mascot-Score**
Staphylococcus aureus				
ANEDESQWNSLLEK	Q5HG77	Transketolase OS=*Staphylococcus aureus* (strain COL)	n.i.	102
VAAWYDNEMSYTAQLVR	Q5HHP5	Glyceraldehyde-3-phosphate dehydrogenase 1 OS=*Staphylococcus aureus*	73	98
WYVIDAEGQTLGR	A7X5B4	50S ribosomal protein L13 OS=*Staphylococcus aureus*	n.i.	68
SQQVWVESVQSSK	A7X659	Immunoglobulin-binding protein sbi OS=*Staphylococcus aureus*	56	59
NEDWQLYTAGK	Q5HCU5	Probable malate:quinone oxidoreductase 2 OS=*Staphylococcus aureus*	35	58
IGETHEGASQMDWMEQEQDR	A6QEJ9	Elongation factor G OS=*Staphylococcus aureus*	n.i.	55
EATGDNWDGLGR	Q2FI18	Probable quinol oxidase subunit 1 OS=*Staphylococcus aureus*	45	53
SSIVVVGTQWGDEGK	P65884	Adenylosuccinate synthetase OS=*Staphylococcus aureus*	n.i.	46
GAFPTWLAPK	A6U2F0	Threonyl-tRNA synthetase OS=*Staphylococcus aureus*	n.i.	46
VVYDITSKPPSTIEWE	A7WY93	GMP synthase [glutamine-hydrolyzing] OS=*Staphylococcus aureus*	45	45
YFDDDDMWNK	A6U2F0	Threonyl-tRNA synthetase OS=*Staphylococcus aureus*	n.i.	33
SVVIAYEPIWAIGTGK	A7WZS8	Triosephosphate isomerase OS=*Staphylococcus aureus*	63	n.i.
LGYDQVLWLDGVEQK	Q5HIC1	Probable branched-chain-amino-acid aminotransferase OS=*Staphylococcus aureus*	84	n.i.
VAAADTIYPFTQAENVWLPNK	P0A0A1	Pyruvate dehydrogenase E1 component subunit beta OS=*Staphylococcus aureus*	38	n.i.
Streptococcus agalactiae				
FSWISTGGGASMELLEGK	A2RCM1	Phosphoglycerate kinase OS=*Streptococcus agalactiae*	n.i.	92
YADTDWGSINSMGLPNK	Q3K2G4	Dihydroorotate dehydrogenase OS=*Streptococcus agalactiae*	84	81
ADIDYAWEEADTTYGK	Q3K3W3	30S ribosomal protein S3 OS=*Streptococcus agalactiae*	n.i.	81
TSVVVVGTQWGDEGK	C0ZA40	Adenylosuccinate synthetase	53	73

		OS=*Streptococcus agalactiae*		
NLGWDIISTGGTK	B5XJ20	Bifunctional purine biosynthesis protein purH OS=*Streptococcus agalactiae*	n.i.	65
AIGPEPWNAAYVEPSR	Q038U2	Glycyl-tRNA synthetase alpha subunit OS=*Streptococcus agalactiae* PE=3 SV=1 -	71	62
GQFGDVWIEFTPNEEGK	Q3JZB5	Elongation factor G OS=*Streptococcus agalactiae*	n.i.	57
WYVVDATDVPLGR	Q1WSC2	50S ribosomal protein L13 OS=*Streptococcus agalactiae*	46	56
VEADANNWETFVVPDNVGGR	Q3K2Y2	Glucose-6-phosphate isomerase OS=*Streptococcus agalactiae*	n.i.	54
GAPEGWVAPVK	Q3K3W2	50S ribosomal protein L16 OS=*Streptococcus agalactiae*	n.i.	52
QQAENLANWAK	Q8E0Z9	Single-stranded DNA-binding protein 3 OS=*Streptococcus agalactiae*	46	51
AFFEIINWNK	P0A4J3	Superoxide dismutase [Mn/Fe] OS=*Streptococcus agalactiae*	n.i.	49
WVNAAEVTEDNIK	Q3K3S2	CTP synthase OS=*Streptococcus agalactiae*	64	n.i.
SSIVVVGTQWGDEGK	P65884	Adenylosuccinate synthetase OS=*Staphylococcus aureus*	53	n.i.

Protein extracts from bacteria were analyzed by LC-MS. Mascot-Scores for the identified tryptophan-containing peptides are given for bacteria grown in the presence of L-Tryptophan (Trp) and bacteria grown in the presence of 1-L-MT. n.i.: not identified. All searches were carried out with methylation of tryptophan residues as variable modifications. No peptide with tryptophan methylation was identified in any sample.
