# Supplementary material for: Influence of Tryptophan Contained in 1-Methyl-Tryptophan on Antimicrobial and Immunoregulatory Functions of Indoleamine 2,3-Dioxygenase
Source: PLoS One. 2012 Sep 13;7(9):e44797. doi: 10.1371/journal.pone.0044797 (PMC3441469; doi:10.1371/journal.pone.0044797)
Supplement: Table S2 — Tryptophan-containing peptides identified by mass spectrometry. Protein extracts from human cells were analysed by LC-MS. Mascot-Scores for the identified tryptophan-containing peptides are given for cells grown in the presence of LTryptophan (Trp) and cells grown in the presence of 1-L-MT. n.i.: not identified. All searches were carried out with methylation of tryptophan residues as variable modifications. No peptide with tryptophan methylation was identified in any sample. (PDF) [file pone.0044797.s006.pdf]

Supplemental table S2. Tryptophan-containing peptides identified by mass spectrometry.

			Trp	1-L-MT
**Sequence**	**Protein accession**	**protein description**	**Mascot Score**	**Mascot-Score**
**Glioblastoma-cells**				
LISWYDNEFGYSNR	P04406	Glyceraldehyde-3-phosphate dehydrogenase OS=*Homo sapiens*	91	n.i.
VVLAYEPVWAIGTGK	P60174	Triosephosphate isomerase OS=*Homo sapiens*	84	n.i.
TVIIEQSWGSPK	P10809	60 kDa heat shock protein, mitochondrial OS=*Homo sapiens*	74	n.i.
VGWEQLLTTIAR	P12814	Alpha-actinin-1 OS=*Homo sapiens*	74	n.i.
DSPSVWAAVPGK	P07737	Profilin-1 OS=*Homo sapiens*	67	68
AWGPGLEGGVVGK	P21333	Filamin-A OS=*Homo sapiens*	64	66
FFPLESWQIGK	P30046	D-dopachrome decarboxylase OS=*Homo sapiens*	60	n.i.
VADIGLAAWGR	P23526	Adenosylhomocysteinase OS=*Homo sapiens*	54	n.i.
SADTLWDIQK	P07195	L-lactate dehydrogenase B chain OS=*Homo sapiens*	54	54
**HeLa Cells**				
LISWYDNEFGYSNR	P04406	Glyceraldehyde-3-phosphate dehydrogenase OS=*Homo sapiens* GN	95	91
VVLAYEPVWAIGTGK	P60174	Triosephosphate isomerase OS=*Homo sapiens*	84	80
DSPSVWAAVPGK	P07737	Profilin-1 OS=*Homo sapiens*	49	71
SADTLWDIQK	P07195	L-lactate dehydrogenase B chain OS=*Homo sapiens*	n.i.	58
FNVWDTAGQEK	P62826	GTP-binding nuclear protein Ran OS=*Homo sapiens*	n.i.	57
FFVGGNWK	P60174	Triosephosphate isomerase OS=*Homo sapiens*	n.i.	47
MPILGLGTWK	P15121	Aldose reductase OS=*Homo sapiens*	50	n.i.
SADTLWDIQK	P00338	L-lactate dehydrogenase A chain OS=*Homo sapiens*	49	n.i.
DDGSAVIWVTFK	Q14019	Coactosin-like protein OS=*Homo sapiens*	70	n.i.
SIQLDGLVWGASK	P29692	Elongation factor 1-delta OS=*Homo sapiens*	67	n.i.
ALPFWNEEIVPQIK	P15259	Phosphoglycerate mutase 2 OS=*Homo sapiens*	53	n.i.
NSSYFVEWIPNNVK	P07437	Tubulin beta chain OS=*Homo sapiens*	52	n.i.

WGDAGAEYVVESTGVFTTMEK	P04406	Glyceraldehyde-3-phosphate dehydrogenase OS=*Homo sapiens*	59	n.i.

Protein extracts from human cells were analysed by LC-MS. Mascot-Scores for the identified tryptophan-containing peptides are given for cells grown in the presence of L-Tryptophan (Trp) and cells grown in the presence of 1-L-MT. n.i.: not identified. All searches were carried out with methylation of tryptophan residues as variable modifications. No peptide with tryptophan methylation was identified in any sample.
